# Direct Nucleation of Hierarchical Nanostructures on Plasmonic Fiber Optics Enables Enhanced SERS Performance

**DOI:** 10.1002/advs.202509947

**Published:** 2025-09-12

**Authors:** Di Zheng, Riccardo Scarfiello, Muhammad Fayyaz Kashif, Liam Collard, Linda Piscopo, Maria Samuela Andriani, Elisabetta Perrone, Concetta Nobile, Massimo De Vittorio, Ferruccio Pisanello, Luigi Carbone

**Affiliations:** ^1^ Istituto Italiano di Tecnologia Center for Biomolecular Nanotechnologies Arnesano Lecce 73010 Italy; ^2^ State Key Laboratory of Radio Frequency Heterogeneous Integration Shenzhen University Shenzhen 518060 China; ^3^ CNR NANOTEC Institute of Nanotechnology Lecce 73100 Italy; ^4^ Dipartimento di Ingegneria Elettrica e delle Tecnologie dell'Informazione Università Degli Studi di Napoli Federico II Napoli 80125 Italy; ^5^ Comprehensive Cancer Centre School of Cancer and Pharmaceutical Sciences King's College London London SE11UL UK; ^6^ Dipartimento di Ingegneria Dell'Innovazione Università del Salento Lecce 73100 Italy; ^7^ IDUN section Department of Health Technology Technical University of Denmark Lyngby DK‐2800 Kgs. Denmark

**Keywords:** branched nanoparticles, hierarchical nanostructures, nanoislands, optical fibers, SERS

## Abstract

An innovative fabrication method is presented to achieve bottom‐up in situ surface‐overstructured Au nanoislands (NIs) with tunable grades of surface coverage, elongation, and branching, directly on micro‐optical fibers for sensing applications. These all‐in‐gold hierarchical nanostructures consist of NIs coated with surface protrusions of various morphologies. They are created in solution using a selective seeded growth approach, whereby additional gold growth is achieved over Au NIs formerly developed on the fiber facet by a solid‐state dewetting approach. The morphology of nanosized surface‐NI overstructuring can be adjusted from multi‐dot‐decorated Au NIs to multi‐arm‐decorated Au NIs. This engineering of optical fibers allows for improved remote surface‐enhanced Raman spectroscopy (SERS) molecular detection. By combining solid‐state dewetting and wet‐chemical approaches, stable in‐contact deposition of surface‐overstructured NIs with the optical fiber solid substrate is achieved, alongside precise control over branching morphology and anisotropy extent. The fiber‐optic probes engineered by surface‐overstructured NIs exhibit outstanding sensing performance in an instant and through‐fiber detection scheme, achieving a remarkable detection limit at 10^−7^ M for the R6G aqueous solution. These engineered probes demonstrate an improved detection limit by one order of magnitude and enhanced peak prominence compared to devices solely decorated with pristine NIs.

## Graphical abstract

An unconventional synthesis strategy enables the direct growth of shape‐tunable nanostructures, from multi‐dot to multi‐arm designs, on silica optical fiber tips. This breakthrough boosts the sensing power of plasmonic fiber‐optic probes and unlocks new possibilities for large‐scale, in situ nanofabrication on silicate‐based substrates, including silica, quartz, and glass.

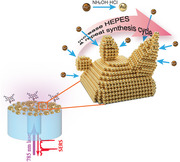

## 1 Introduction

Enabling fiber‐optic probes with surface‐enhanced Raman scattering (SERS) sensing ability can extend SERS applications to scenarios where sampling is challenging and minimal invasiveness to the system is required, such as probing in vivo biological tissue,^[^ ^1^, ^2^ ^]^ site‐specific study for living cells,^[^ ^3^, ^4^ ^]^ and on‐site environmental monitoring.^[^ ^5^, ^6^, ^7^ ^]^ Plasmonic nanoparticles are the most popular candidate to be integrated into the fiber tip since: i) they support local plasmon resonances (LPRs), generating strong near‐field enhancement,^[^ ^8^, ^9^, ^10^ ^]^ ii) they can actively interact with guided modes through near‐field coupling,^[^ ^11^, ^12^ ^]^ iii) bottom‐up fabrication approaches compatible with both flat and curved fiber facets can enable wide‐surface and high throughput fabrication.^[^ ^13^, ^14^, ^15^, ^16^, ^17^ ^]^ A particularly interesting type of plasmonic nanoparticle in this context is the branched nanoparticle, an anisotropic nanostructure that exhibits multiple LPRs with distinct resonant wavelengths, optimal performance at longer wavelengths, and strong electromagnetic fields near the branches.^[^ ^18^, ^19^, ^20^ ^]^ The increased surface area provides extended sites to interact with analytes, contributing to enhanced SERS performance compared with simple particle‐shaped structures.

Efforts have been made to obtain multi‐branched nanostructures (MBNSs) on the fiber facet to fabricate SERS‐active fiber probes, and the currently employed methods are mainly based on electrostatic self‐assembly (ESA). ESA requires first synthesis of the target batch of MBNSs with high purity, then using a molecular linker to functionalize the silica or glass surface to obtain heterogeneous charges between the fiber and the MBNS’ surfaces to realize nanoparticles immobilized on the fiber surface.^[^ ^21^, ^22^, ^23^ ^]^ To circumvent this time‐consuming batch pre‐synthesis, ligand exchange, and self‐assembly steps, a powerful synthetic methodology is represented by bottom‐up in situ substrate growth based on wet‐chemical synthesis to form morphologically controlled nanostructures directly on the supporting substrates.^[^ ^24^ ^]^ For MBNSs, the growth methods compatible with oxide substrates were demonstrated by first attaching colloidally prepared seeds to a substrate so that subsequent in situ overgrowth into anisotropic shapes.^[^ ^25^, ^26^ ^]^ This method still involves seed pre‐synthesis, surface functionalization, and self‐assembly steps. It shows that without prefixed seeds, the in situ growth of MBNSs can be difficult as it cannot take advantage of the native ability of the substrates, such as hydrogels with amide and carboxyl/hydroxyl groups^[^ ^27^ ^]^ and organosilica, hydridosilica, or other silicone‐based polymers that have Si‐H groups,^[^ ^28^, ^29^ ^]^ to reduce metal salts. Top‐down lithography methods can be used to pattern seeds on the substrates,^[^ ^30^, ^31^ ^]^ despite requiring a time‐consuming lift‐off and transfer process. The overall low production efficiency hinders the scalability of the device and translation into real settings.

The scientific community, therefore, requires more general and high‐throughput methods to enable the exploitation of the interesting electromagnetic properties of morphology‐elaborated nanostructures on substrates ready for applications, such as fiber optics for molecular sensing. To the best of our knowledge, there is a lack of literature on scalable in‐situ nano‐structuring on fiber facets with variable morphologies of Au nanostructures for remote SERS detection. This work is meant to fill this gap, reporting a method that can be expanded to every glass substrate.

We introduce a novel bottom‐up in situ growth approach for fabricating Au composition‐homogeneous and morphology‐tunable hierarchical nanostructures straightforwardly on the end facet of multi‐mode silica optical fibers. This approach enables precise morphologically controlled nanostructuring of the fiber facet, using a two‐phase process. First, we used a solid‐state dewetting approach to nucleate Au nanoislands (NIs) uniformly distributed on the fiber facet, with an average diameter of ≈50 nm and an inter‐NIs distance of ≈30 nm. In the second phase, we used a colloidal wet‐chemical synthesis method to promote homogeneous nucleation and growth of additional Au over the surface of preexisting NI seeds. This process was facilitated by the rapid reduction of Au^3+^ ions using hydroxylamine (NH_2_OH·HCl) in the presence of varying concentrations of HEPES.^[^ ^32^, ^33^ ^]^ The wet‐chemical procedure can be repeated twice; in the first stage, multi‐dot‐decorated Au NIs (MDot‐NIs) are produced, whereby the density of coverage can be adjusted according to the reagent concentration. Whereas, multi‐arm‐decorated Au NIs (MArm‐NIs) develop when the wet chemical process is performed twice; in the latter case, considerable elongation and branching are favored. The whole synthetic procedure allows for the control of the shape of the resulting complex nanostructures and the extent of dot‐to‐rod elongation attuned to the chemical conditions. This process prevents the formation of free particles in solution within the experimental timeframe. We demonstrate a clear shape transition from surface dot‐rich nanostructures to branched nanostructures, promoting a shift from a thermodynamic to a kinetic growth regime. Whether the chemical conditions enabled the control of the density of Au over‐deposition on pre‐existing NI seeds, the number of wet‐chemical synthetic steps triggered the shape transition. The two‐step wet‐chemical synthetic procedure allowed the synthesis of composition‐homogeneous variable morphology hierarchical nanostructures (from MDot‐NIs to MArm‐NIs), henceforth generally referred to as hierarchical nanoislands (HNIs), densely, uniformly, and stably distributed over the fiber facet. Moreover, multiple fibers can be simultaneously exposed to the same in‐situ chemical conditions, making this process scalable, robust, and reproducible. The optical fibers engineered with HNIs exhibit superior SERS enhancement outperforming pristine only‐NIs‐engineered fibers; this improvement is evident in both direct‐facet and through‐fiber excitation schemes when the surface is functionalized with BT molecules. Meanwhile, the limit of detection (LOD) experiment conducted on an aqueous solution of R6G demonstrates that the optical fibers engineered with HNIs reduce the LOD by one order of magnitude compared to only‐NI‐engineered fibers in an instant and through‐fiber detection scheme. Among all the developed HNIs, the MDot‐NIs‐engineered fiber devices achieved the lowest LOD of 10^−7^ M. Our method not only demonstrates its effectiveness in enhancing the SERS performance of plasmonic fiber‐optic probes but also enables large‐scale in situ engineering of hierarchical nanostructures on silicate‐based substrates (including silica, quartz, and glass), offering a cost‐effective, scalable, and efficient strategy for fabricating highly sensitive SERS structures.

## 2 Results

**Figure 1:**
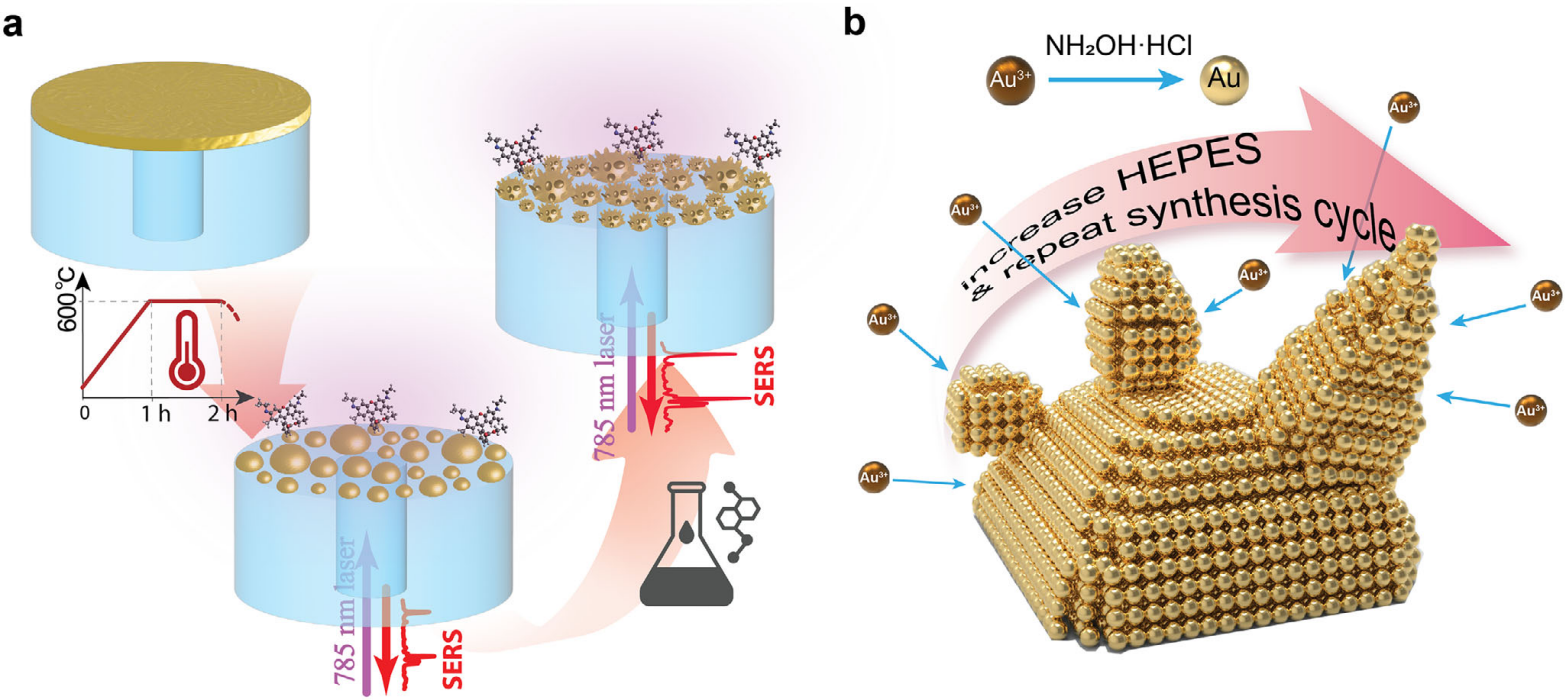
The sketch illustrates the method to fabricate HNIs on the fiber facets. a) A solid‐state dewetting technique is at first used to convert a thin layer of a gold film deposited across the fiber facet into hemispherical NIs that subsequently serve as seeds over which additional gold nanostructures are deposited via a HEPES‐driven wet‐chemical approach. b) By adjusting the concentration of HEPES and the number of wet‐chemical steps, the morphology of Au nanostructures achieved across the surface of NIs can be changed from dot‐ to arm‐shaped.

The overall fabrication process is illustrated in **Figure** **1**. The two‐phase process combines a solid‐state dewetting stage with multiple wet‐chemical colloidal steps. The first solid‐state high‐temperature (600 °C for 1 h) dewetting stage^[^ ^14^ ^]^ allows generating a uniform distribution of hemispherical Au NIs starting from a 5 nm thin film e‐beam‐evaporated onto the facet of core/cladding fibers (Figure 1a). Thereafter, NIs distributed over the fiber tip serve as seeds to self‐catalyze the over‐deposition of additional gold in the shape of dots through a HEPES‐driven wet‐chemical synthesis process. The density of gold over‐deposition above each NI can be adjusted according to the wet‐chemical conditions. Additionally, the elongation and branching of the lately formed dot‐shaped gold nanodomains can further be promoted by repeating the wet‐chemical growth process (Figure 1b).

**Figure 2:**
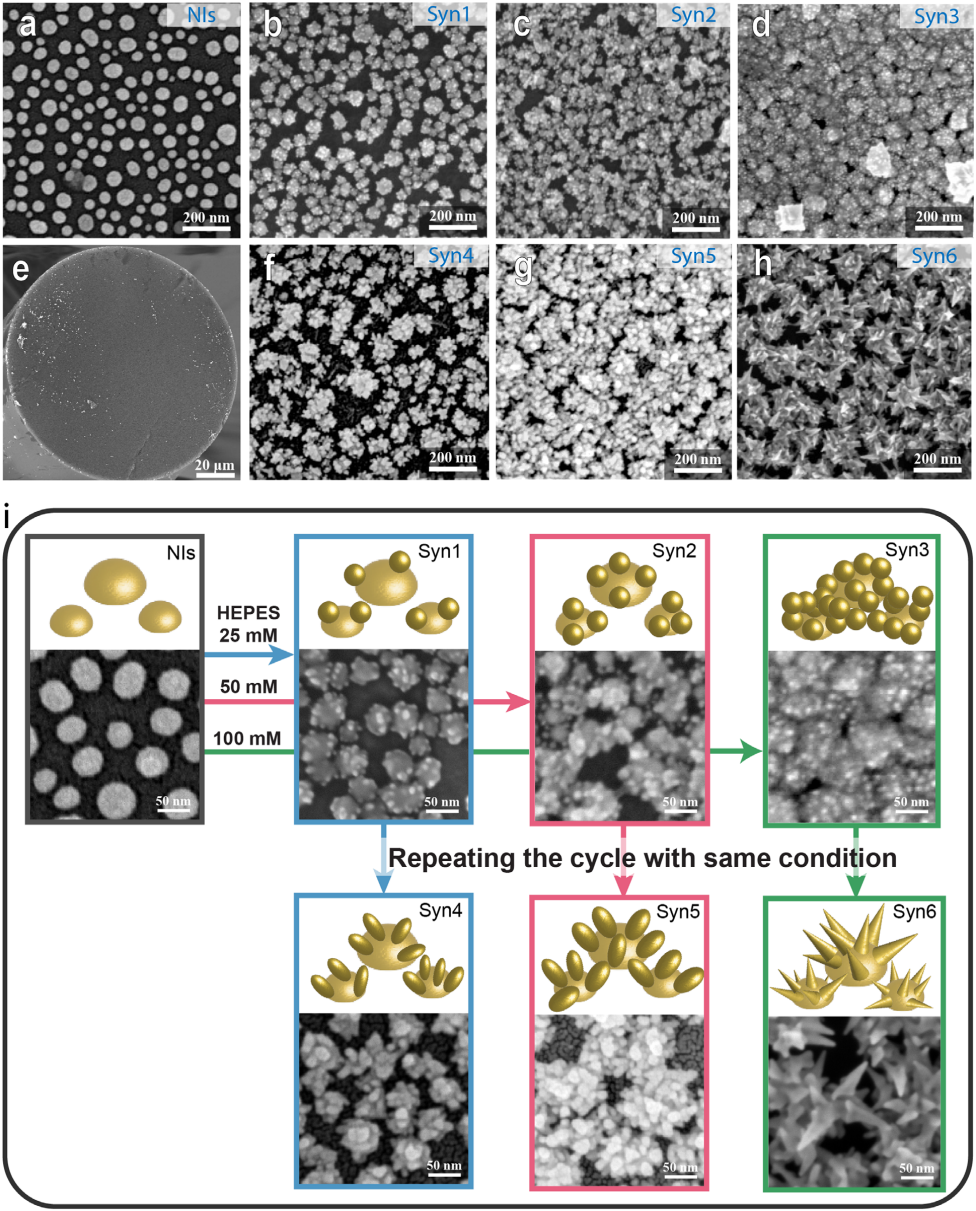
Gallery of SEM overviews of HNIs obtained by variable chemical‐driven synthesis protocols. a) The morphology of pristine solid‐state dewetted NIs as seeds prefixed on the fiber. b–d) SEM morphologies of samples (labeled as Syn1‐, Syn2‐, and Syn3‐HNIs) were obtained using a single wet‐chemical synthesis protocol with HEPES concentrations of 25, 50, and 100 mm in an 8 mL reaction media, respectively. e) The representative fiber facet of HNIs exhibits a uniform surface status across the entire facet after synthesis. f–h) SEM morphologies of samples (labeled as Syn4‐, Syn5‐, and Syn6‐HNIs) were obtained using two sequential wet‐chemical synthesis protocols with HEPES concentrations of 25, 50, and 100 mm in an 8 mL reaction media, respectively. The other synthetic conditions were unchanged. i) Sketch illustrations and respective high‐magnification SEM images depicting the morphological evolution of Au hierarchical nanostructures.

All the fabrication results are presented in **Figure** **2**; The pristine NIs seeds are shown in Figure 2a. SEM images illustrating the overview of the fiber facet (Figure 2e) and morphological evolution of the resulting Au nanostructures developed over the optical fiber tips are shown in Figure 2b–d,f–h (large view SEM images are provided in Figures S1 and S2, Supporting Information for all HNIs). In detail, the nucleated NIs, with an average diameter of 50 nm and an inter‐NI spacing of 30 nm, uniformly cover the entire fiber facet (Figure 2a); these will serve as seeds to facilitate subsequent material nucleation via seeded growth. The tip of the NI‐decorated optical fiber is immersed within an 8 mL mixture of NH_2_OH·HCl (0.625 mm) and HEPES (25, 50, or 100 mm) for three separate experiments. In each case, 1 mL of HAuCl_4_·3H_2_O (1.75 mm) aqueous solution is added dropwise to the reaction media, at room temperature (RT), while stirring. The pH is set to 7.4, whereas the HEPES concentration in the solution is changed. Generally, in these conditions, HEPES serves as a shape‐directing agent and NH_2_OH·HCl as a reducing agent of gold ions for the development of dot‐like Au nanodomains. It is accepted that HEPES buffer is thermodynamically capable of working as both a reducing agent (due to cation free radical on the tertiary amine of the piperazine group)^[^ ^32^, ^33^, ^34^ ^]^ as well as a shape‐directing agent with its sulfonic acid group.^[^ ^34^, ^35^, ^36^, ^37^, ^38^, ^39^, ^40^ ^]^ Considering the established rate of Au addition, the time of growth is ≈20 min (see experimental details). Under conditions of very low HEPES concentration 25 mm spherical gold dots nucleate onto preformed NIs, resulting in the formation of MDot‐NIs (Figure 2b). By doubling the concentration to 50 mm, while always keeping constant the amount of gold, the number of dot‐like domains decorating each NI increases (Figure 2c); this trend continues, reaching a densely packed dot deposition at the highest HEPES concentration of 100 mm (Figure 2d). For the wet‐chemical experiments just described, a thermodynamic regime of growth prevails, mostly forming dot‐shaped nanodomains reasonably nucleating over lattice defects exposed by the original NIs. At the very low time of growth of a wet‐chemical step (20 min), the reducing ability is yielded by NH_2_OH·HCl and HEPES, the latter being weakly reducing at these rather dilute concentrations.^[^ ^35^ ^]^ Nanodots nucleate and grow in such a chemical environment.

The sulfonate groups of the HEPES molecules preferentially bind to Au surfaces, exposing the free hydroxyl groups. This interaction leads to selective chemical stabilization of specific surfaces, resulting in crystallographic elongation. As the concentration of HEPES increases, the chemospecific stabilization becomes stronger. At higher concentrations of HEPES, the molecules self‐assemble into structures with long‐range order, creating a soft template that drives the formation of highly elongated, anisotropic gold nanoparticles.^[^ ^40^ ^]^

Multiple cycles of wet‐chemical Au deposition can be conducted to facilitate the anisotropic development of dot domains according to the HEPES concentration. Indeed, by repeating the Au reduction in solution over fiber tips engineered by MDot‐NIs, elongation and branching of dot domains occur. Under conditions of 25 mm HEPES concentration, spherical Au dots evolve into low‐aspect‐ratio nanorods (Figure 2f) that become more elongated by doubling the HEPES concentration to 50 mm (Figure 2g). With an even higher HEPES concentration of 100 mm, longer and branched arms develop generating MArm‐NIs (Figure 2h). This kinetic process outcome can be explained by an enhanced reducing capacity of the wet‐chemical environment despite the diluted concentrations of HEPES; it can be rationalized on these bases. i) Seed‐assisted reduction: small dots earlier developed represent favorable sites of nucleation on which supplemental Au deposition occurs rather than forming new domains from scratch.^[^ ^41^ ^]^ ii) Surface‐catalyzed reduction of Au^3+^: the presence of small dots strengthens the reducing ability of NH_2_OH·HCl;^[^ ^31^, ^32^, ^41^ ^]^ the greater the number of dots, the faster the reduction. This latter kinetic effect limits the formation of in‐solution free particles because of a reductant‐assisted autocatalytic deposition of Au triggered by pre‐existing Au domains. In analogy with the electroless Au plating deposition, Au growth continues until the catalytic substrate is removed from the solution or the gold ion concentration is depleted. This autocatalytic Au over‐deposition is significantly enhanced upon highly‐dot‐decorated NIs. iii) Shape‐directing effect of HEPES molecules: these passivate and stabilize Au surfaces, driving the formation of anisotropic and branched nanostructures as they generate templating structures with a long‐range order.^[^ ^35^ ^]^ All this evidence helps to explain the kinetic regime in the fast experimental time scale adopted and rules out possible Ostwald ripening events. A thermodynamically driven growth regime would induce a redistribution of Au atoms within the pre‐existing surfaces, resulting in a smoother round‐edge morphology because of atom migration from energetically unfavorable sites (such as sharp edges or tips) to decrease the surface average curvature. This process would be slower compared to the autocatalytic growth.^[^ ^42^ ^]^ By replicating these procedures several times, more densely packed nanostructures are grown, and the Au overgrowth reduces the original NI inter‐distances.

The replication of a ligand‐mediated, kinetic‐driven process, rather than simply increasing the overall precursor concentration, was employed to limit spontaneous nanoparticle formation (secondary nucleation) through seedless nucleation in solution and to promote heterogeneous nanoparticle overgrowth on the optical fiber. Indeed, to favor the in situ anisotropic development of gold NIs instead of promoting secondary nucleation in the solution, the experimental strategy of multiple injections was employed rather than solely increasing the precursor concentration in a single growth step.^[^ ^24^, ^31^ ^]^ Moreover, based on the catalytic role of NH_2_OH·HCl in facilitating the reduction of Au^3+^ in the presence of gold surfaces, in situ growth was conducted with an excess of the reducing agent to further prevent secondary nucleation of free nanoparticles in the solution. Previous studies have shown that HEPES can produce gold nanostars (AuNS) across a wide buffering range (pH 6.9−8.2).^[^ ^43^ ^]^ Therefore, we selected and maintained the pH at 7.4, which is slightly below the pKa (7.55) of the nitrogen atom in the piperazine ring near the sulfonic group (also referred to as N2). This deprotonated form of the nitrogen can form a weak covalent bond with gold, ensuring that HEPES functions actively yet non‐invasively during the synthesis process. Based on these considerations, all other reaction parameters were kept constant, apart from HEPES concentration.

It is worth noting that nucleation of Au nanostructures on optical fibers does not occur in the absence of dewetted NI seeds preexisting on the fiber tip (Figure S3, Supporting Information). Figure 2i sketches the above‐described process of Au deposition over NIs along with the high‐magnification SEM images of the detailed structures. To assist the reader in referencing each sample, from this point onward, the developed structures reported in Figure 2b–d,f–h will be referred to as Syn1‐, Syn2‐, Syn3‐, Syn4‐, Syn5‐, and Syn6‐HNIs, respectively.

**Figure 3:**
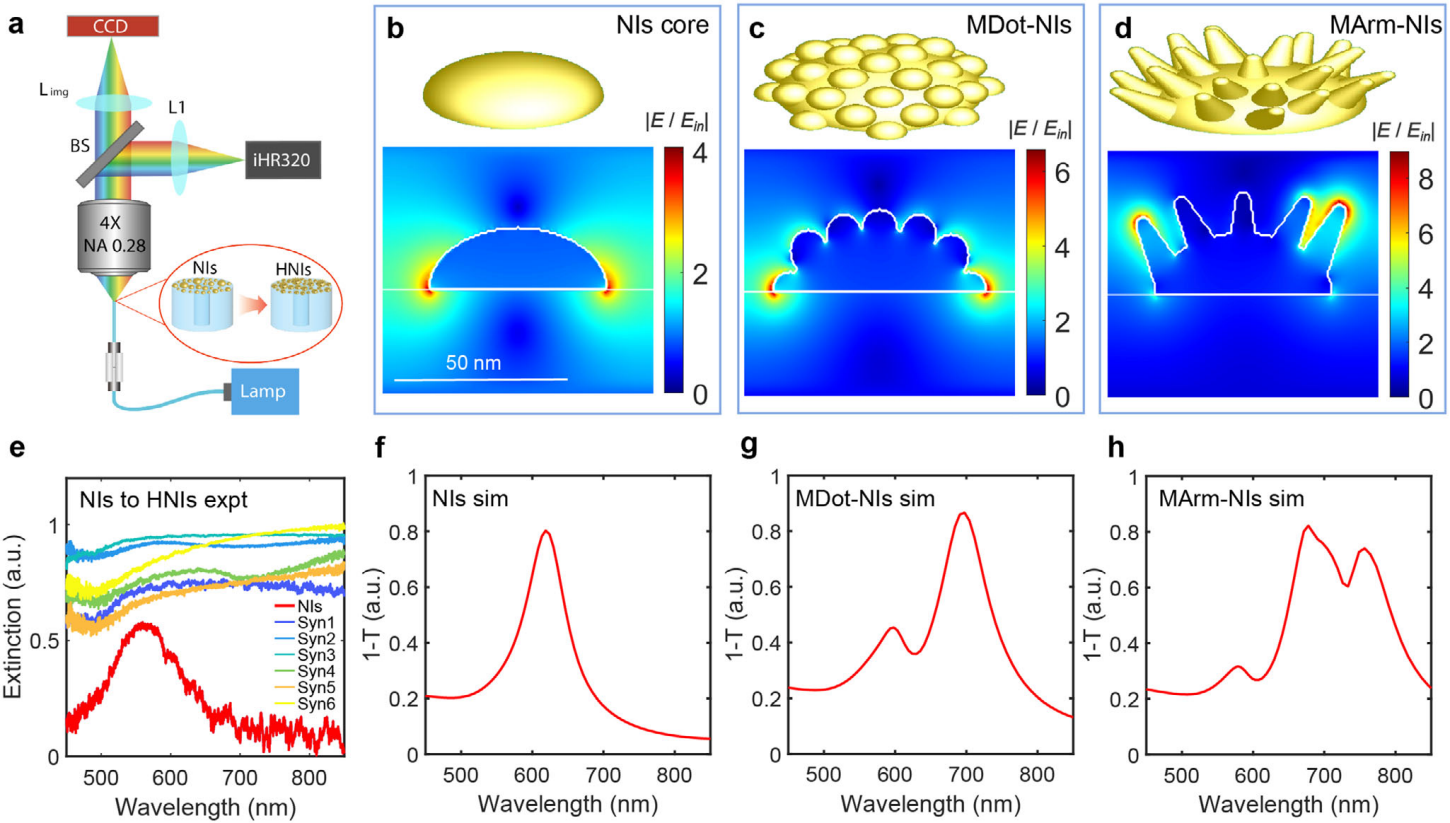
The electromagnetic response for NIs and HNIs. a) A schematic illustration of the optical setup used for extinction measurements (BS – 50:50 beam splitter, iHR320 – Horiba spectrometer, CDD – charge‐coupled device). b–d) Simulation models for NIs, MDot‐NIs, and MArm‐NIs are arranged in a square periodic configuration. The core NIs have a diameter of 50 nm and a height of 15 nm, with a particle lattice periodicity of 76 nm. MDot‐NIs’ and MArm‐NIs’ models are derived from the NI model by adding 10 nm diameter gold nanospheres and nanocones (56 nm long, 20 nm wide at the base) embedded in the NIs core, respectively, across their surfaces. The field enhancements are obtained by normal incidence and 785 nm plane wave excitation. e) Extinction spectra of fibers with facets decorated by NIs (red) and HNIs (gradient from blue to yellow). f–h) Spectral responses of square periodic NIs, MDot‐NIs, and MArm‐NIs, are represented using the coefficient (1 ‐ T), where T is the transmittance of the system at normal incidence.

To further understand the electromagnetic response of the obtained geometry‐complex nanostructures, we performed extinction measurements using the configuration illustrated in **Figure** **3**a. White halogen light was guided to the plasmonic fiber facet through a fiber patch cord. The transmitted light from the fiber tip was collected by an Olympus objective (4X, NA = 0.28) and subsequently directed into a spectrometer for analysis (details in Experimental Section). The extinction results are shown in Figure 3e. The resonance transitions from single resonance peaks in NIs to broader, flattened, and increased extinction profiles in HNIs‐engineered fibers. We also conducted a statistical morphological analysis to classify the obtained HNI structures. This analysis aimed to extract representative geometric parameters for constructing a simplified electromagnetic model allowing for a better understanding of the nanostructures (details provided in Figure S4, Supporting Information). Assuming normal distributions, the fitting results show that the average diameters increase from ≈50 nm for NIs to 60 nm for the Syn1‐HNIs sample, and the diameter distribution broadens following the overgrowth of branches. For spike structures, we measured perpendicular spikes to maximize statistical accuracy, finding an average spike length of 56 nm and a bottom width of 20 nm (detailed results in Figure S5, Supporting Information). Using these parameters, we developed a simplified numerical model consisting of a periodic square array of gold hemispheres for NIs (diameter of 50 nm, height of 15 nm, and periodicity of 76 nm, parameter details are outlined in the Methods section). By decorating the surface of NIs with nanospheres (10 nm in diameter) and nanocones, we constructed models for MDot‐NIs and MArm‐NIs. These models enable isolation and analysis of the effects of closely packed HNIs while avoiding computationally intensive calculations. The 3D models and near‐field cross‐sectional distributions for NIs and HNIs in one unit cell are shown in Figure 3b–d. The HNIs exhibit higher field enhancement at 785 excitations compared to NIs, with MArm‐NIs showing the greatest field enhancement at the branch tip regions. The corresponding spectral responses (expressed as 1 – T, where T is the transmittance of the system at normal incidence) are presented in Figure 3f–h. These results demonstrate a transition from a single resonance peak in NIs to multiple resonances in HNIs. The final optical properties of HNIs are thus found to be anisotropic and dependent on the morphological feature overstructuring the surface of original NIs. While the simulated model effectively avoids computationally demanding calculations and successfully captures the general trend of resonance transitions, from a single peak to multiple peaks as structural complexity increases, it does not fully reflect the structural variability observed in the experimental system. In experiments, nanostructures exhibit a high degree of heterogeneity in terms of size, shape, protrusion number, and arrangement, which leads to broader and more complex spectral features than those predicted by the uniform simulation. Notably, the similar extinction spectra observed across all synthesized structures in Figure 3e likely result from overlapping plasmon resonance modes, caused by the structural disorder and morphological inhomogeneity that are inherent in the synthesis process.

**Figure 4:**
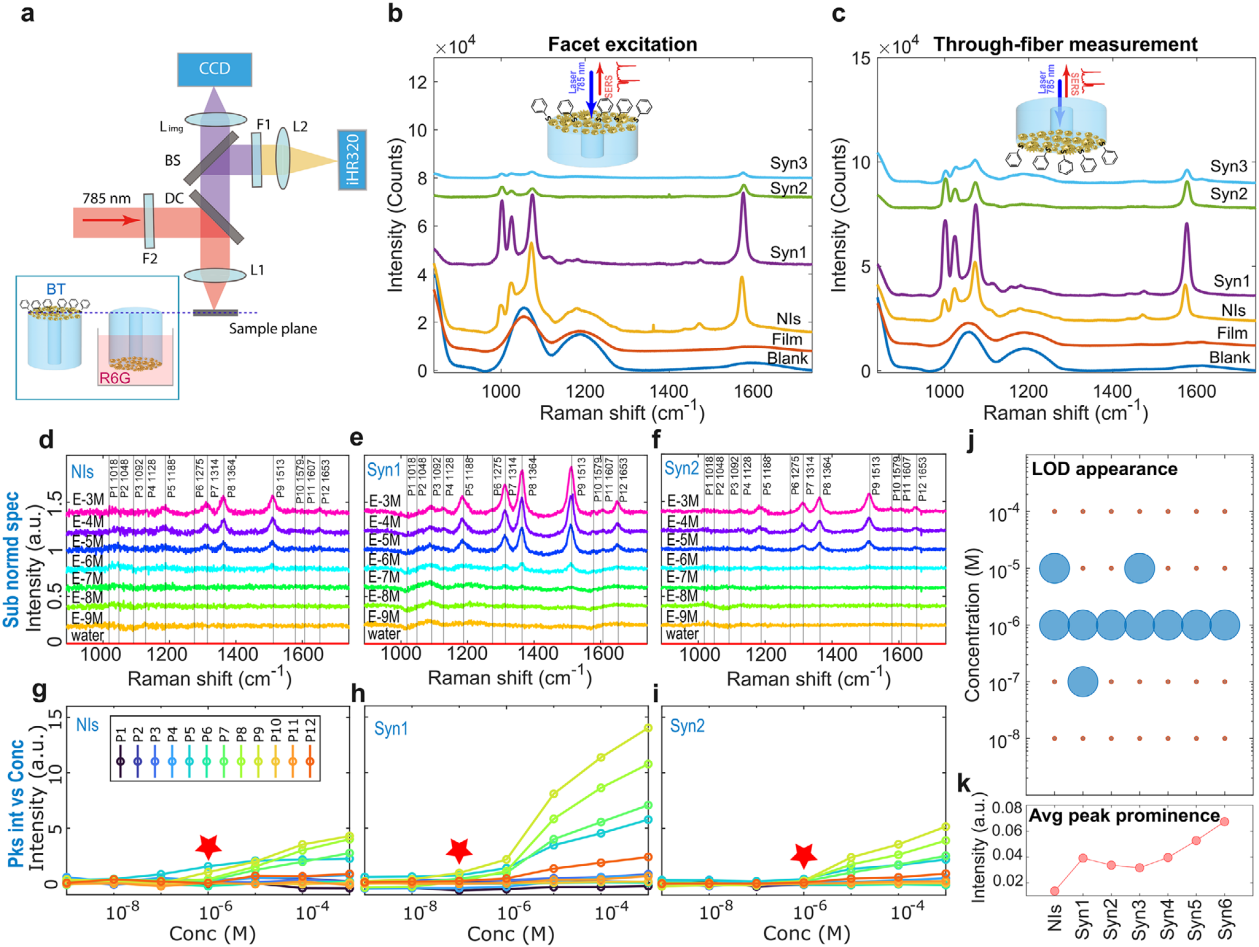
SERS characterizations for HNI fibers. a) The schematic illustration for the optical configuration for SERS measurement; the fibers can be configured with direct facet excitation and through‐fiber excitation (DC‐long pass dichroic, BS‐beam splitter, F1‐notch filter, iHR320‐spectrometer, and CCD‐charge coupled device). b,c) The facet excitation and through‐fiber excitation measurement with BT molecules functionalized on the fiber facet. All the fibers underwent the same functionalization procedures using the BT molecule. d–f) The selected R6G LOD SERS spectra of fibers functionalized with NIs and Syn1/Syn2‐HNIs; complete spectral sets of the reference fibers (blank and film‐covered), and synthesized fibers are provided in Figures S6 and S7 (Supporting Information); the spectra were normalized to the silica peak at 1055 cm^−1^, and the silica background was subtracted (the silica background was taken as the normalized spectra measured in water). The spectra sets have been vertically offset for clarity. The vertical lines mark the 12 R6G peak positions. g–i) The peak intensities (integral of peak areas) against the concentrations, and the red stars mark the LOD for each type of fiber. j) A summary of LOD appearance for NIs and all the HNIs fibers; the LOD is marked as the lowest concentration when any of the R6G signature peaks of 1314, 1364, and 1513 cm^−1^ become observable in the subtracted spectra. k) The average peak prominence values for NIs and all HNIs‐decorated fibers; each data point is based on an average of two measurements per condition.

The SERS performance of the fabricated probes was initially benchmarked by benzenethiol (BT) molecules functionalized on the gold surface, aiming at evaluating the different performance of fibers hosting different types of nanostructures (blank, NIs, and Syn1‐/Syn2‐/Syn3‐HNIs). Then, direct‐facet excitation and through‐fiber excitation of the probes were tested (optical setup in **Figure** **4**a). The results in Figure 4b,c show that the Syn1‐HNI fiber can generate most of the SERS signal under both excitation conditions. An increase in the density of dots over NIs correlates with a decrease in signals for both Syn2‐ and Syn3‐HNIs. This outcome suggests that the Syn1‐HNIs are more effective as SERS‐active nanostructures when integrated over the fiber tip. The more separated nanostructures in the Syn1‐HNIs case are more efficient in generating hotspots than the more connected or continuous film‐like structures in the Syn2 and Syn3 cases. Studies also have shown that dense film assemblies of Au nanostars do not necessarily create more efficient hotspots or increase the SERS enhancement.^[^ ^20^, ^44^, ^45^ ^]^

Most application scenarios for SERS‐active fiber probes involve remotely detecting specific chemicals in surrounding media; LOD experiments can provide insights into the performance of the fiber‐optic probes. Thus, a set of fibers was prepared with various facet functionalizations: blank, film‐covered, NIs, Syn1‐ to Syn6‐HNIs. These were tested using the chemically stable analyte R6G in solution. A range of R6G aqueous solutions with concentrations from 10^−9^ to 10^−3^ M was prepared, and through‐fiber SERS measurements were conducted using a custom‐built Raman microscope (details provided in the Experimental Section). For each fiber, measurements started with the lowest concentration solution and progressed incrementally to the highest concentration, using the same single fiber for each concentration step. Each spectrum was recorded immediately after fully immersing the fiber tip in the aqueous solution, which remained submerged throughout the exposure period. The representative measured spectra at different concentrations are reported in Figure 4d–f. These spectra were obtained by first normalizing to the silica peak at 1055 cm^−1^ and then subtracting the silica background; the probe's silica background for subtraction was measured in water. The blank and film‐covered fibers showed minimal detection of R6G peaks across all tested concentrations (see more details in Figure S6, Supporting Information). In contrast, the NI and all synthesized HNI fibers exhibited sensitive SERS detection at low concentrations. For a detailed comparison, we integrated the areas of 12 specific peaks (1018, 1048, 1092, 1128, 1188, 1275, 1314, 1364, 1513, 1579, 1607, and 1653 cm^−1^) as a measure of peak intensity and plotted this against the concentrations. The selective results for NIs, Syn1‐ and Syn2‐HNI fibers are reported in Figure 4g–i (more detailed results in Figure S7, Supporting Information). The data shows that Syn1‐HNI fibers had the lowest LOD at 10^−7^ M. Notably, the peaks’ prominence varied with different HNI morphologies, even when the LOD remained the same. We analyzed a statistical dataset comprising 15 fibers (including NIs and different synthesis parameters of HNI fibers), with an average of 2 fibers examined for each synthesis condition. The results in Figure 4j illustrate the LOD appearance for NIs and all six synthesis conditions of HNI fibers. For the NI fibers, the LOD ranges from 10^−6^ to 10^−5^ M, whereas, the LOD for Syn1‐HNI fibers falls within 10^−7^ to 10^−6^ M. Most of the other synthesis conditions demonstrate a LOD of 10^−6^ M, except for Syn3‐HNI fibers, which show a LOD between 10^−6^ and 10^−5^ M. Furthermore, the peak prominence from the statistical analysis is summarized in Figure 4k. It shows that all the synthesized HNI fibers exhibit significantly higher peak prominence compared to pristine NI fibers. The peak prominence for each spectrum set of R6G LOD is determined by integrating the areas under all 12 peak intensities relative to concentration, as represented in Figure 4g–i. The average values for fibers with the same synthesis conditions were used to depict the peak prominence intensity in Figure 4k.

## 3 Discussion and Conclusion

Our two‐phase in situ growth method, which combines solid‐state dewetting with wet‐chemical synthesis, the latter under seed‐mediated and surfactant‐assisted growth control, enables the scalable fabrication of HNIs directly on optical fiber facets. This two‐phase approach effectively addresses the limitations of traditional techniques, such as ESA and top‐down lithography. By leveraging Au surface‐catalyzed reduction mediated by hydroxylamine and HEPES, we achieved tunable HNI morphologies ranging from MDot‐ to MArm‐NIs, with dense and uniform coverage. The extent of elongation and branching in the dot‐to‐arm shape transition can be controlled. The cross‐section for the excitation of these complex multifaceted nanostructures increases compared to the original NIs.^[^ ^19^ ^]^ This method is scalable, allowing for the simultaneous processing of multiple fibers under identical conditions, and it can be adapted for use with different glass substrates. The HNI‐functionalized fibers demonstrated superior SERS performance compared to pristine NI fibers as well as higher surface area. Notably, the multi‐dot‐shaped Syn1‐HNI fiber probes achieved the lowest LOD of 10^−7^ M for R6G. All types of HNI fibers generally show better signal prominence compared to pristine NI fibers (Figure 4k), indicating increased field enhancement for HNIs. However, it is worth noting that while field enhancement may improve signal generation, it does not necessarily lead to a lower LOD. Several factors can affect the LOD appearance when resolving molecular signatures through fibers.^[^ ^17^ ^]^ In our case, we should consider three main factors: i) the ability to generate the SERS signal, ii) the ability to collect the SERS signal (occurring through the optical fiber), and iii) the relative strength of the collected SERS signal compared to the silica background.

For different morphologies, the increased field enhancement leads to a stronger SERS signal, with the signal predominantly generated at the hot spots where the electromagnetic field is most concentrated. As shown in Figure 3b–d, hot spots for NIs and MDot‐NIs are primarily located near the interface between the substrate and nanostructures. In contrast, MArm‐NIs exhibit hot spots at the tips of the branches, elevated above the substrate. This morphological difference influences the collection of the SERS signal through the fiber (from the modal perspective, the coupling efficiency between guided modes and the plasmonic resonance). When nanostructures are positioned at the tip of the fiber and excited by light, they create hot spots at specific spatial regions determined by the nanostructure geometry. When target molecules are situated within these hot spots, a Raman dipole can be induced. The nanostructures then act as optical antennas, converting the evanescent molecular field into propagating far‐field radiation. In this process, the plasmonic modes largely govern the radiation pattern, which typically exhibits a wide angular distribution.^[^ ^46^, ^47^ ^]^ To enhance device performance, it is crucial to direct the Raman dipole radiation into the fiber, enhancing the coupling efficiency between fiber's guided modes and the plasmonic resonance, rather than allowing it to dissipate into the surrounding environment. Although the NIs and MDot‐NIs demonstrate comparatively weaker near‐field enhancement than MArm‐NIs (as shown in Figure 3b–d), their hot spots are located very close to the fiber and nanostructure interface. This spatial configuration likely results in a larger fraction of the Raman emission being coupled back into the fiber, thereby improving the efficiency of SERS signal collection.^[^ ^48^ ^]^ Furthermore, as the number of dots/branches (higher density of Au deposition over NIs) increases and their size grows around the NI core, more material accumulates on the fiber surface, and the structures become more interconnected. This creates optical conditions (including local effective refractive index and local metal confinement) that increase the sensitivity to silica's Raman background, reducing the overall analyte/background signal ratio. As a result, the relative strength of the molecular signals diminishes, obscuring signatures at low concentrations. Ultimately, the probe's performance results from the combined effects of hot spot intensity and positioning, structural density, and background signal interference.

In our study, the MDot‐NIs of Syn1‐HNI fibers, characterized by their well‐defined dot‐shaped protrusions and well‐separated distribution, achieved the best performance, reaching the lowest LOD at 10^−7^ M among all tested morphologies. This superior performance is attributed to a well‐balanced combination of hot spot intensity, spatial positioning, structural density, and minimal background interference. Future optimizations of this approach could target several key areas: first, increasing the inter‐particle distance between NIs to minimize the interconnection of branches. The solid‐state dewetting technique provides significant tunability by modifying deposition parameters, such as initial film thickness and deposition rate.^[^ ^17^, ^49^ ^]^ Second, fine‐tuning the wet‐chemical synthesis to achieve shorter and sharper tips throughout the NI core will enhance field concentration and improve signal collection. Additionally, integrating these probes with other sensing technologies, such as fluorescence or electrochemical sensors, could expand their potential applications.

In conclusion, this study presents a breakthrough in the fabrication of SERS‐active fiber‐optic probes using an innovative, scalable, bottom‐up in situ substrate growth method. By controlling the complex geometry of Au composition‐homogeneous hierarchical nanostructures through the selective growth of dot‐ or arm‐shaped outgrowths over NIs, we achieve significant improvements in SERS sensitivity. Lower detection limits and enhanced signal‐to‐noise ratios demonstrate this. This approach enables the creation of high‐performance optical sensors that can be seamlessly transformed into tapered fibers,^[^ ^14^, ^17^, ^50^ ^]^ enhancing the efficiency of holographic SERS endoscopic imaging.^[^ ^15^ ^]^ Moreover, it shows great potential for practical applications, including in vivo biological monitoring and real‐time environmental sensing.

## 4 Experimental Section

### Fabricating HNIs on the Fiber Facet

The standard multimode silica optical fibers (Thorlabs, FG050LGA, NA = 0.22, Low‐OH, Ø50 µm Core) were used for flat fiber fabrication. As a preparation step, all the fibers were put in an acetone bath for 30 min to remove the acrylate jacket. The fibers were cut with a fixed length of 7.5 cm. Then, a manual fiber cleaver (Thorlabs, XL411) was used to cut one side of the fiber to obtain a smooth top flat fiber facet. Then fibers were fixed on a batch mount in an e‐beam evaporator (Thermionics laboratory, inc. e‐GunTM), with their top surface normally aligned to the gold source in the crucible. A 5 nm thick gold film was evaporated on the fiber facet; the evaporation rate was kept at 0.2 Å·s^−1^, with chamber pressure <6 × 10^−6^ mbar. When the evaporation procedure finished, the fibers were detached from the mount and arranged in a ceramic bowl without any adhesive for thermal annealing in a muffle furnace (Nabertherm B180). The furnace was set to gradually ramp up from RT to 600 °C with a rate of 10 °C min^−1^ and held at 600 °C for 1h, then allowed to cool ambiently to RT. After the NIs had been fabricated, it started with the wet chemical treatment. A surfactant‐assisted methodology^[^ ^51^ ^]^ was selected, where HEPES acts as a shape‐directing agent, NH_2_OH·HCl operates as a reducing agent for Au^3+^ to Au^0^ in the presence of Au nanodomain^[^ ^32^ ^]^ and previously deposited Au NIs as a catalytic seeding surface. A proper volume (200–800 µL) of HEPES (1 M, with pH adjusted at 7.4 with NaOH) and 50 µL of NH_2_OH·HCl (100 mm) water solution was dispersed in 7.8–7.2 mL ultrapure water, respectively, in order to obtain 8 mL as the final volume in each reaction media. The resulting concentrations in the reaction media for HEPES are 25, 50, and 100 mm, and for NH_2_OH·HCl was 0.625 mm. NIs‐functionalized fibers were then immersed in the solution. Then, 35 µL of HAuCl_4_·3H_2_O (50 mm) solution was dispersed in 950 µL of water, loaded in 3 mL syringes, and slowly cast at room temperature in the gently stirred reaction media with a syringe pump (parameters: diameter 6 mm, rate 50 µL·min^−1^). After the injection, the fiber was rinsed three times by dipping it in a flask with clean H_2_O for 20 s. The setup allows the contemporary processing of several fibers during the same process. Moreover, the same fiber can be exposed to multiple injection processes. Regarding the HEPES regulation, a series of synthesis reactions was typically conducted within the same batch of pre‐synthesized nanoisland seeds. This provides a stable foundation for the subsequent overgrowth of hierarchical nanostructures. During the synthesis process, the consistency and reproducibility of the samples were consolidated by carefully controlling key synthetic parameters such as the specific volumes derived from freshly prepared stock solutions, the timing and volume of injections using a syringe pump, and maintaining the RT at 25 ± 1 °C. These measures ensured satisfactory reproducibility between different batches, even when multiple fibers were treated in solution simultaneously. Moreover, negligible sample‐to‐sample variation was detected independently of the number of fibers contemporarily treated in solution. This was explained by the fact that we worked constantly in excess of hydroxylamine and Au^3+^. The HEPES (4‐(2‐hydroxyethyl)‐1‐piperazineethanesulfonic acid, C₈H₁₈N_2_O_4_S), NaOH, NH_2_OH·HCl, and HAuCl_4_·3H_2_O were purchased from Sigma Aldrich.

### The Extinction Measurements for NIs and HNIs Fibers

The extinction spectra were measured with a transmission configuration using a home‐built microscope. Briefly, a halogen lamp with an SMA connector was used to excite the fiber through a fiber patch‐cord connection, which delivered the broadband light through the NIs and HNIs functionalized fiber facet. The transmitted light was collected with a 4X objective (Olympus XLFluor 4x/340, NA = 0.28), and then directed to a spectrometer (Horiba iHR320) with an achromatic doublet (Ø25.4 mm, f = 100 mm). The spectrometer was equipped with a 300 l·mm^−1^ grating and synapse EMCCD for the spectra acquisition. To subtract the actual extinction spectrum of NI and HNI‐functionalized fibers (*Ext*_NIs_), the transmission spectrum of NI and HNI fibers (*T*_NIs_) was corrected by a blank fiber transmission spectrum (*T*_blank_) with relation *Ext*_NIs_ = (*T*_blank_ − *T*_NIs_)/*T*_blank_, to exclude the systematic response.

### The Morphological Analysis for NIs and HNIs

SEM images were acquired with the FEI Helios Nanolab 600i Dual Beam system for the morphological analysis. The SEM image acquisitions were usually conducted after sputtering a thin layer of gold on the NI fiber’ surface, while some chemically synthesized HNI fibers can have enough conductivity for SEM imaging without any sputtering. Home‐developed MATLAB programs were used for NI morphological analysis. By tracing object boundaries, the NI number count and the coverage situations were obtained. The coverage rate (*C_1_ =* 33.34%) and the areas of physical occupation (*S*_1_) were extracted, and the diameters were then determined by $D_{1}=2\times \sqrt{S_{1}/\pi }$, the result is shown in the histogram in Figure S4 (Supporting Information). The average height *(H*_1_ *=* 15 nm) of the NIs can be computed by *H*_1_ = *Thk/C*_1_, where *Thk =* 5 nm is the initial film deposition thickness. For the Syn1‐HNI analysis, as the HNIs are more branching each other, only the coverage rate (*C*_2_ = 53%) was extracted by tracing object boundaries using gray level, then, a manual method was used to extract the diameters of Syn1‐HNIs and included nanoparticles as many as possible within the obtained SEM images to form statistics by ImageJ. For the MArm‐NIs (Syn6‐HNIs), the perpendicular spikes were measured to maximize statistical accuracy. It was found that the average spike length was 56 nm, and the bottom width was 20 nm. Detailed results can be found in Figure S5 (Supporting Information).

### Electromagnetic Simulations

The geometric parameters obtained from morphological analysis, including NI average diameters (*D*_1_), height (*H*_1_), and coverage rate (*C*_1_), were used to build up a simplified numerical model using the finite difference time domain (FDTD) method (Ansys‐Lumerical), to emphasize the effect of the interparticle distances and reduce the computational cost. Thus, the NI pattern was represented with an Au droplet‐like structure (to mimic the NI shape) arranged in a periodic square array, having a diameter of 50 nm and a maximum height of 15 nm. The period of the unit cell was set as 76 nm. The C_1_ was defined as the ratio between the gold disk area and the square unit cell area. The C_1_ and D_1_ are known from the morphological analysis. The same square periodic arrangement was used as NIs for HNIs. As the diameters of NIs and Syn1‐HNIs are ≈50–60 nm, thus nanospheres of 10 nm diameter embedded into the NI core were used to form the HNIs. For MArm‐NIs, the nanocones (with a length of 56 nm and a bottom width of 20 nm) were randomly integrated with NIs to mimic the branched structure. By simulating 3D time‐harmonic Maxwell's equations, the results in Figure 3 were obtained. The particle sits on an infinite dielectric substrate with a refractive index *n* = 1.4. Optical constants of gold were obtained from Ref.[52] The spectral response was obtained by scanning the source's wavelength from 400 to 900 nm, while field enhancement maps were generated at 785 nm, a widely employed wavelength for Raman inspection of biological samples.

### SERS Characterizations on the Fibers

To prepare the fibers for optical characterizations, the unstructured side of fabricated fibers was connected to metallic ferrules with a diameter of 1.25 mm and underwent a manual polishing process. For the optical measurements, a home‐built Raman microscope was used for characterization; further details can be found in the previous publication.^[^ ^17^ ^]^ Briefly, the linear polarized free space laser of 785 nm continuous wavelength was coupled into a meter‐long fiber patch cord to launch the laser into the excitation path of the Raman microscope, and the resulting excitation laser delivered to the sample was scrambled into a speckle pattern. The collimated laser beam filled the back aperture of the focus lens L1 (aspheric, Ø25.0 mm, f = 20 mm), resulting in a light spot of 50 µm in diameter, which matches the fiber core size. For the facet excitation, the fibers were configured with a proximal end facing the focus lens with a power of 6 mW. In the through‐fiber excitation, the fibers were configured with the distal end facing the focus lens with a power of 10 mW; the laser was injected over the full angular acceptance of the fiber (NA = 0.22) to recruit most of the propagating modes. The Raman signals were then separated from the pump laser using a dichroic mirror (DC: Semrock, LPD02‐785RU‐25) and a long‐pass razor‐edge filter (F1: Semrock, LP02‐785RU‐25). Then, the signal was routed to a spectrometer (Horiba iHR320). The Raman measurements were performed with a slit at 200 µm and a 600 l·mm^−1^ (blaze 750 nm) grating. Spectra were recorded on a SYNAPSE CCD cooled to −50 °C. All the raw spectra were treated with baseline correction (ALS). In the through‐fiber SERS measurements. The spectra acquisition time was 60 s for all measurements. For the BT molecule functionalized fiber measurements, all reference fibers were immersed in a 6 mm methanol solution of BT molecule for 6.5 h. Afterward, the fibers were rinsed by stirring them in a cup of clean methanol solution for 10 min. This rinsing process was repeated three times, using fresh methanol solution each time, to ensure successful monolayer functionalization. Both BT (benzenethiol, C_6_H_5_SH) and R6G (Rhodamine 6G, C_28_H_31_N_2_O_3_Cl) were purchased from Merck KGaA.

## Conflict of Interest

The authors declare no conflict of interest.

## Supporting information



Supporting Information

## Data Availability

The data that support the findings of this study are available from the corresponding author upon reasonable request.
