# Supplementary material for: Direct Nucleation of Hierarchical Nanostructures on Plasmonic Fiber Optics Enables Enhanced SERS Performance
Source: Adv Sci (Weinh). 2025 Sep 12;12(42):e09947. doi: 10.1002/advs.202509947 (PMC12622514; doi:10.1002/advs.202509947)
Supplement: Supplementary file 1 — Supporting Information [file ADVS-12-e09947-s001.docx]

**Supporting Information for**

**Direct Nucleation of Hierarchical Nanostructures on Plasmonic Fiber Optics Enables Enhanced SERS Performance**

*Di Zheng^1,2,*,†^, Riccardo Scarfiello^1,3,†^, Muhammad Fayyaz Kashif^1,4^, Liam Collard^1,5^, Linda Piscopo^1,6^, Maria Samuela Andriani^1,6^*, *Elisabetta Perrone^3^, Concetta Nobile^3^, Massimo De Vittorio^1,6,7,*^, Ferruccio Pisanello^1,*,†^, Luigi Carbone^1,3,*,†^*

*^1^ Istituto Italiano di Tecnologia, Center for Biomolecular Nanotechnologies, Arnesano, Lecce, 73010, Italy*

*^2^ State Key Laboratory of Radio Frequency Heterogeneous Integration, Shenzhen
University, Shenzhen 518060, China*

*^3^ CNR NANOTEC, Institute of Nanotechnology, Lecce, 73100, Italy*

*^4^ Dipartimento di Ingegneria Elettrica e delle Tecnologie dell'Informazione, Università Degli Studi di Napoli Federico II, 80125 Napoli, Italy*

*^5^ Comprehensive Cancer Centre, School of Cancer and Pharmaceutical Sciences, King’s College London, London, SE11UL, UK*

*^6^ Dipartimento di Ingegneria Dell’Innovazione, Università del Salento, Lecce 73100, Italy*

*^7^ IDUN section, Department of Health Technology, Technical University of Denmark, DK-2800 Kgs. Lyngby, Denmark*

*^*^ Authors to whom correspondence should be addressed:* [*zhengdi@whu.edu.cn*](mailto:zhengdi@whu.edu.cn.com)*,* [*massimo.devittorio@iit.it*](mailto:massimo.devittorio@iit.it)*,* [*ferruccio.pisanello@iit.it*](mailto:ferruccio.pisanello@iit.it), *luigi.carbone@cnr.it*

***^†^*** *These authors contributed equally to this work*


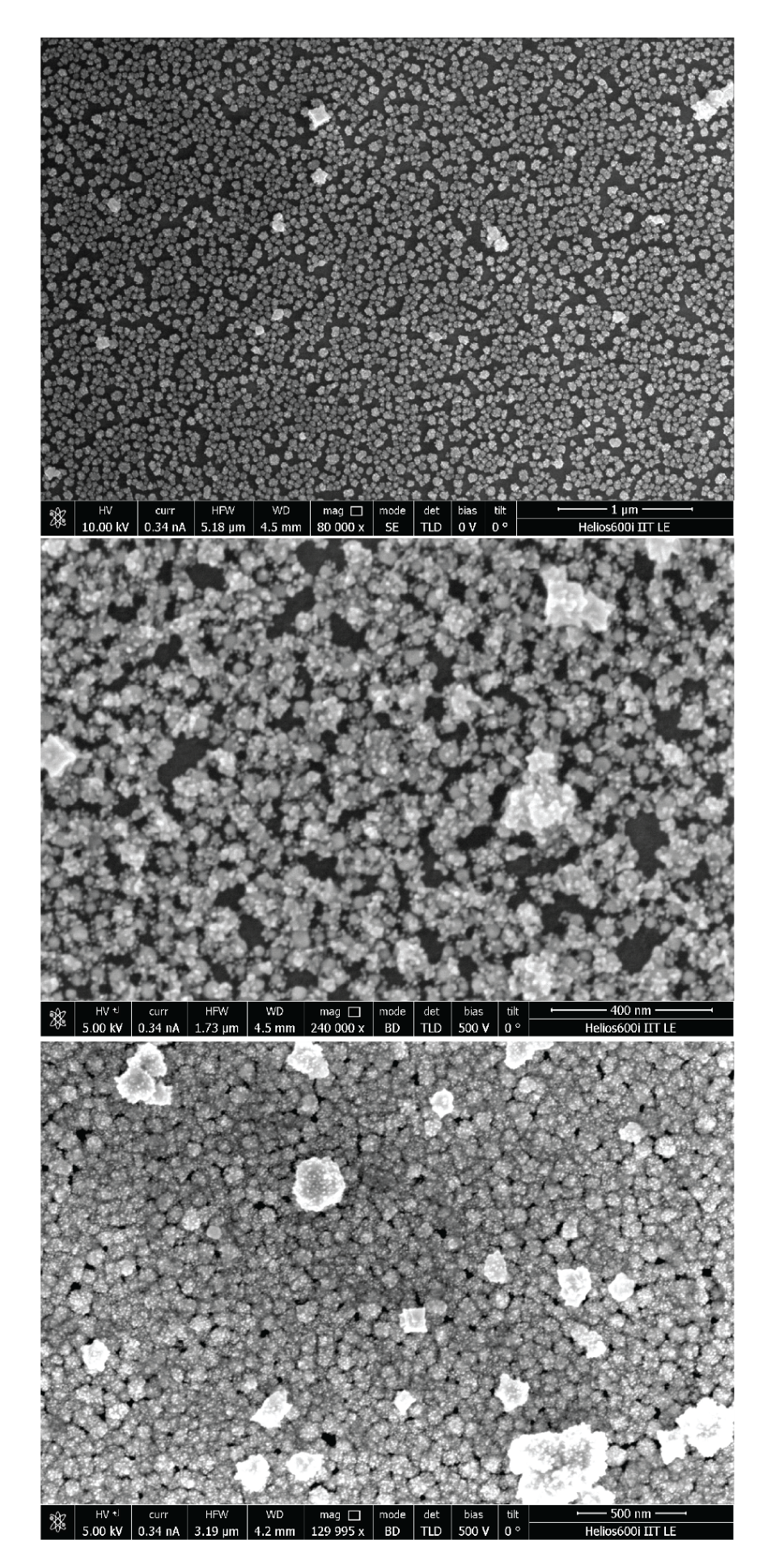


**Figure S1** SEM overview of HNIs obtained through a single wet-chemical synthesis protocol; from top to bottom are Syn1-, Syn2-, and Syn3-HNIs respectively.


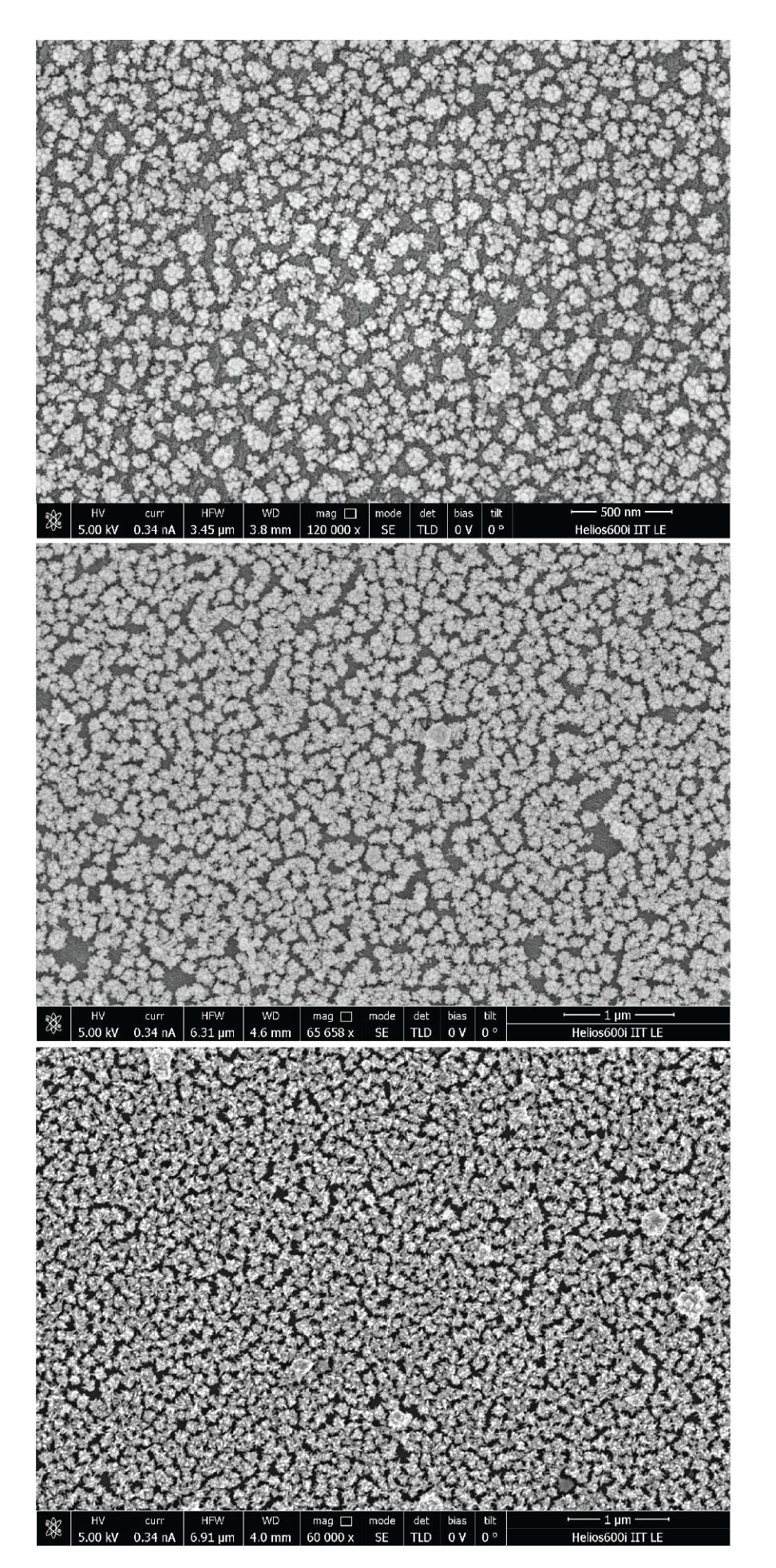


**Figure S2** SEM overview of HNIs obtained through two sequential wet-chemical synthesis protocol; from top to bottom are Syn4-, Syn5-, and Syn6-HNIs respectively.

*
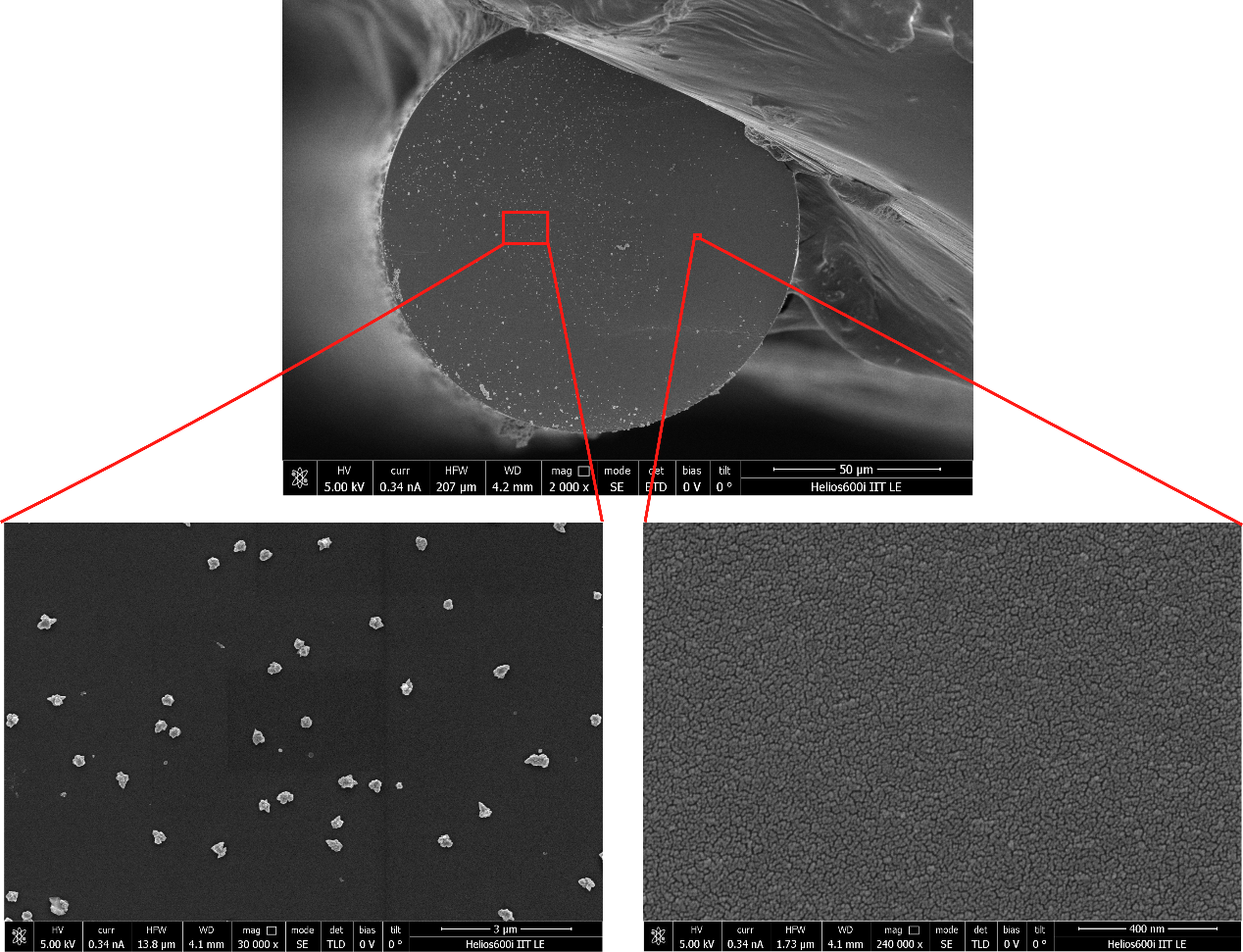
****Figure S3*** *SEM overview of the unseeded synthesized fiber tip. The red square indicates the surface area highlighted in the zoomed-in SEM images at higher magnifications: 30,000× (left) and 240,000× (right). The large impurities observed on the left side of the fiber tip (approximately 300 nm in diameter and spaced several micrometers apart) are most likely residual contaminants from the fiber fabrication or handling process, rather than gold-related synthesized particles. The otherwise clean background further supports that no seeded gold nanoparticles were formed on the unseeded fiber tip.*


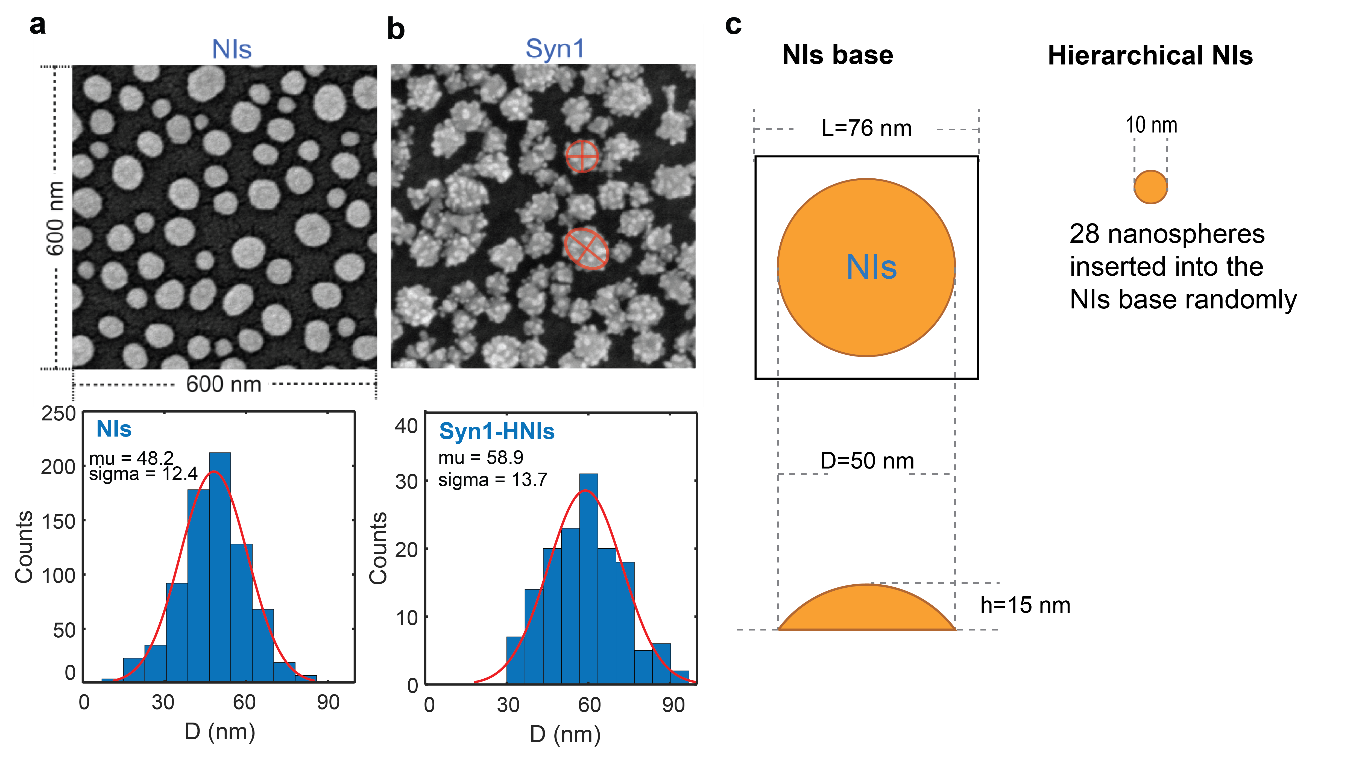
***Figure S4*** *(a) Morphology of the NIs (upper panel) and corresponding diameter distribution (lower panel). The NIs were extracted using gray-level difference analysis. (b) Morphology of the Syn1-HNIs (upper panel) and diameter analysis (lower panel). Diameters were determined by averaging measurements from all manually assessed HNIs, with two-axis measurements taken for each particle based on the profile. Two examples are highlighted with red circles and crosses in the SEM image. (c) Square periodic model for the NIs based on average diameter and coverage rate. The Syn1-HNIs model was constructed by randomly inserting 28 nanospheres into the base of the NIs.*

*
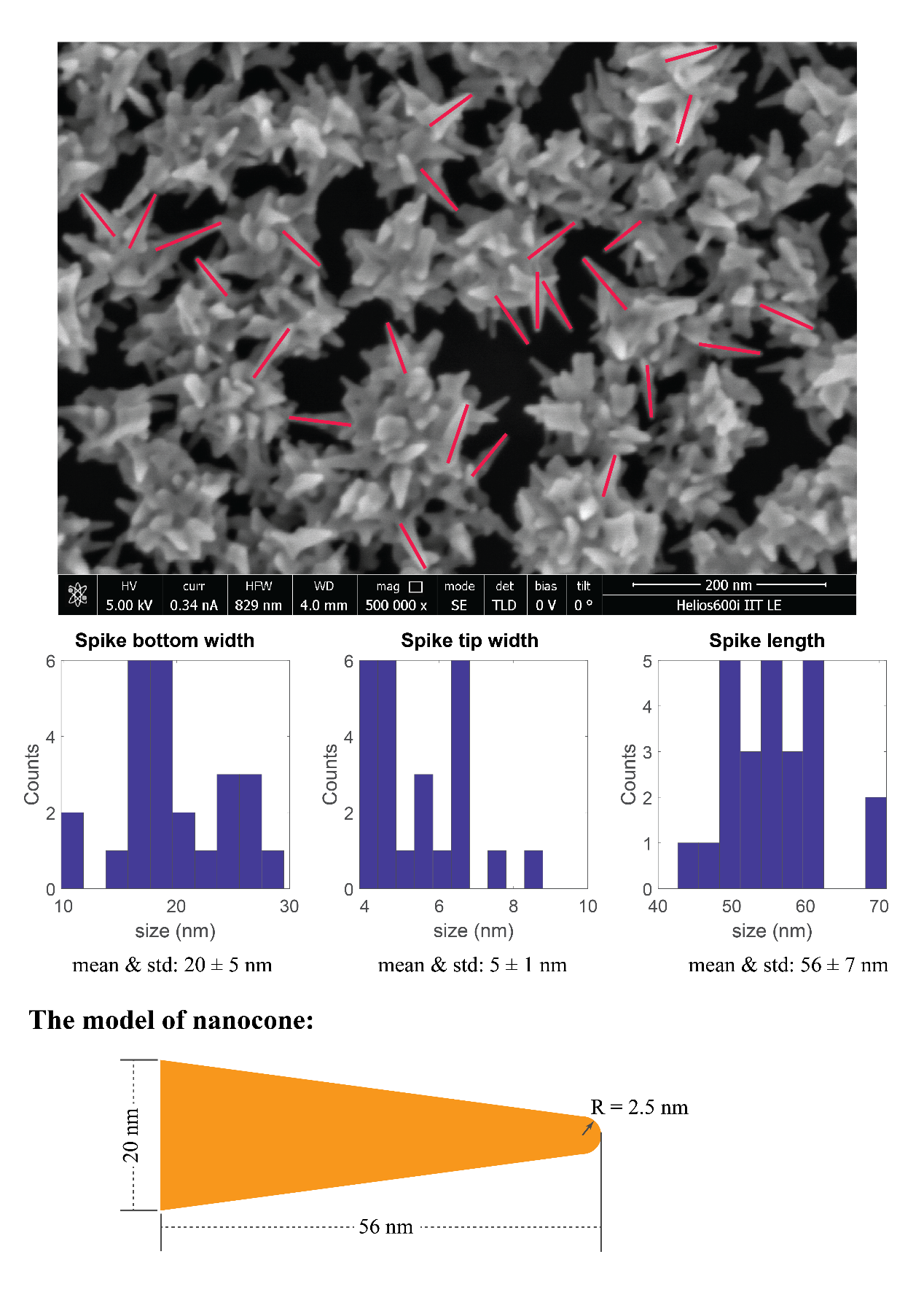
****Figure S5*** *Morphology analysis of Syn6-HNIs. Perpendicular spikes were manually measured, as indicated by the red lines in the SEM image. The bar graphs display the measured dimensions of the spikes. Using the average geometric parameters extracted from the SEM image, the spikes were modeled as nanocones for simulation, with a base length of 20 nm, a side length of 56 nm, and a tip rounding of 2.5 nm.*

*
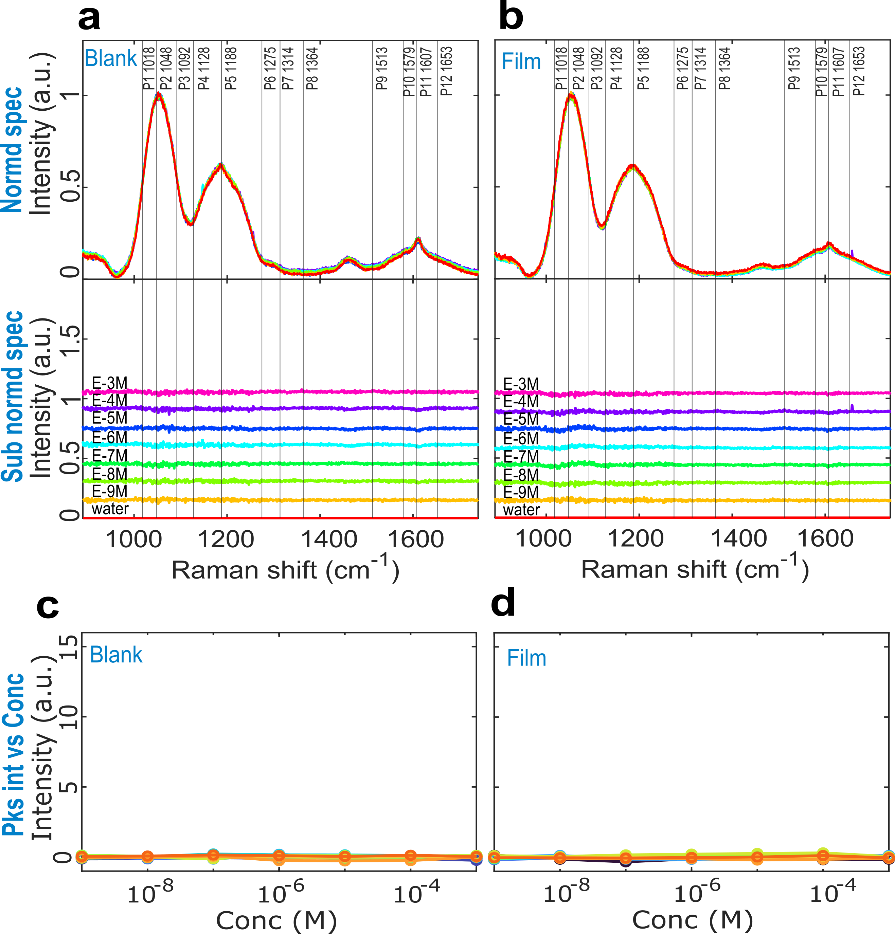
*

***Figure S6*** *The R6G LOD SERS spectra of blank and gold film-covered fibers. (a and b) The original spectra (up) and subtracted spectra (bottom) at different concentrations; the spectra were normalized to silica peak at 1055 cm^-1^, and subtracted silica background (the silica background was taken as the normalized spectra measured in water). The spectra sets have been vertically offset for clarity. The vertical lines mark the 12 R6G peak positions. (c to d) The peak intensities (integrate peak areas) against the concentrations.*

*
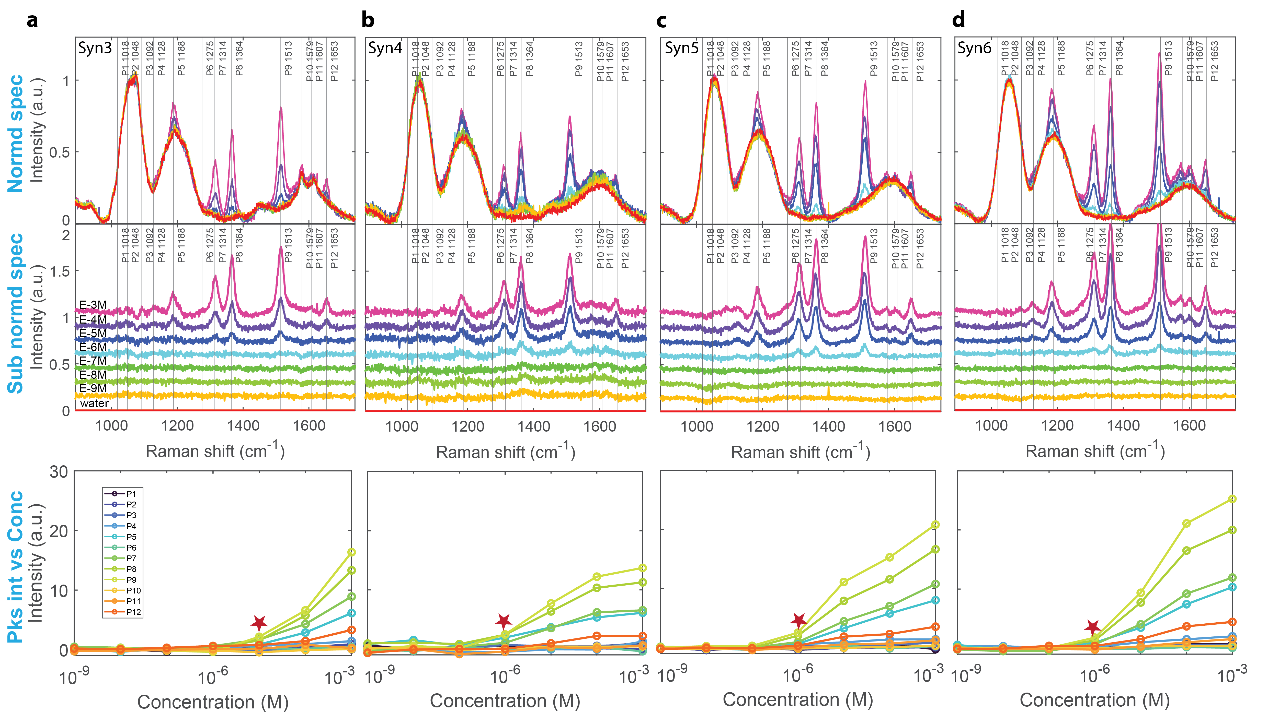
****Figure S7*** *(a-d) Representative SERS spectra of R6G LOD for Syn3-, Syn4-, Syn5-, and Syn6-HNI fibers. The upper panel displays the normalized original spectra and the background-subtracted spectra, while the bottom panel shows the peak intensities (integrated peak areas) as a function of concentration.*
